# *Klebsiella pneumoniae* infection biology: living to counteract host defences

**DOI:** 10.1093/femsre/fuy043

**Published:** 2018-11-18

**Authors:** José A Bengoechea, Joana Sa Pessoa

**Affiliations:** Wellcome-Wolfson Institute for Experimental Medicine, Queen's University Belfast, Belfast BT9 7BL, UK

**Keywords:** *Klebsiella*, innate immunity, virulence

## Abstract

*Klebsiella* species cause a wide range of diseases including pneumonia, urinary tract infections (UTIs), bloodstream infections and sepsis. These infections are particularly a problem among neonates, elderly and immunocompromised individuals. *Klebsiella* is also responsible for a significant number of community-acquired infections. A defining feature of these infections is their morbidity and mortality, and the *Klebsiella* strains associated with them are considered hypervirulent. The increasing isolation of multidrug-resistant strains has significantly narrowed, or in some settings completely removed, the therapeutic options for the treatment of *Klebsiella* infections. Not surprisingly, this pathogen has then been singled out as an ‘urgent threat to human health’ by several organisations. This review summarises the tremendous progress that has been made to uncover the sophisticated immune evasion strategies of *K. pneumoniae*. The co-evolution of *Klebsiella* in response to the challenge of an activated immune has made *Klebsiella* a formidable pathogen exploiting stealth strategies and actively suppressing innate immune defences to overcome host responses to survive in the tissues. A better understanding of *Klebsiella* immune evasion strategies in the context of the host–pathogen interactions is pivotal to develop new therapeutics, which can be based on antagonising the anti-immune strategies of this pathogen.

## Teaser

The human pathogen *Klebsiella pneumoniae*: a master puppeteer of manipulation of our body to counteract our defences.

## INTRODUCTION

*Klebsiella pneumoniae* was first described by Carl Friedlander in 1882 as a bacterium isolated from the lungs of patients who had died from pneumonia (Friedlander 1882). *Klebsiella* species are ubiquitously found in nature including water, soil and animals, and they can colonise medical devices and the healthcare environment (Podschun and Ullmann 1998; Podschun *et al.*^2001^). *Klebsiella* species are considered opportunistic pathogens colonising mucosal surfaces without causing pathology; however, from mucosae *Klebsiella* may disseminate to other tissues causing life-threatening infections including pneumonia, UTIs, bloodstream infections and sepsis (Paczosa and Mecsas 2016). *K. pneumoniae* infections are particularly a problem among neonates, elderly and immunocompromised individuals within the healthcare setting (Magill *et al.*^2014^). This organism is also responsible for a significant number of community-acquired infections worldwide (Ko *et al.*^2002^). Defining features of these infections are the ability to metastatically spread and their significant morbidity and mortality (Paczosa and Mecsas 2016). *Klebsiella* strains associated with these infections are regarded as hypervirulent, and recent epidemiological studies indicate that these strains share specific genetic characteristics (Holt *et al.*^2015^).

*K. pneumoniae* is gaining attention due to the rise in the number of infections and the increasing number of strains resistant to antibiotics. More than a third of the *K. pneumoniae* isolates reported to the European Centre for Disease Prevention and Control were resistant to at least one antimicrobial group, the combined resistance to fluoroquinolones, third-generation cephalosporins and aminoglycosides being the most common resistance phenotype (European Centre for Disease Prevention and Control Antimicrobial resistance (EARS-Net) 2018). Furthermore, *Klebsiella* species are a known reservoir for antibiotic-resistant genes, which can spread to other Gram-negative bacteria. In fact, many of the antibiotic-resistant genes now commonly found in multidrug-resistant organisms were firstly described in *Klebsiella*. Very few therapeutic options are left for patients infected with multidrug-resistant *K. pneumoniae* with additional resistance to carbapenems, and are often limited to combination therapy and to colistin. Alarmingly, recent studies have recognised that several *K. pneumoniae* virulent and multidrug-resistant clones have access to a mobile pool of virulence and antimicrobial resistance genes (Holt *et al.*^2015^; Lam *et al.*^2018^; MC Lam *et al.*^2018^), making then possible the emergence of a multidrug, hypervirulent *K. pneumoniae* clone capable of causing untreatable infections in healthy individuals. Unfortunately, there are already reports describing the isolation of such strains (Zhang *et al.*^2015^, ^2016^; Gu *et al.*^2018^; Yao *et al.*^2018^). Despite its clinical relevance, our understanding of *K. pneumoniae* pathogenesis contains considerable gaps, thereby making a compelling case to better understand its infection biology to design new strategies to treat *Klebsiella* infections.

Recent excellent reviews have covered the epidemiology of *Klebsiella*-triggered infections, the mechanisms of resistance to antibiotics and the description of some of the virulence factors of this pathogen (Paczosa and Mecsas 2016; Navon-Venezia, Kondratyeva and Carattoli 2017; Martin and Bachman 2018). This review focuses on the complex interaction between *Klebsiella* species and the innate immune system, and summarises our understanding of *Klebsiella* anti-immune strategies. Although central to the infection biology of multidrug-resistant pathogens such as *Klebsiella*, the repertoire of their adaptations to the human immune system are generally overlooked. However, the co-evolution of these bacteria in response to the challenge of an activated immune system has made them formidable pathogens. As will be apparent in this review, *Klebsiella* can no longer be considered only as a stealth pathogen. *Klebsiella* has developed an array of systems that ‘surgically strike’ key regulators and effectors of the host immune system, placing this pathogen as a master puppeteer controlling several anti-immune evasion systems to overcome host responses to survive in the tissues.

## INNATE IMMUNE DEFENCES AGAINST BACTERIAL INFECTIONS

Infection can be viewed as a consequence of specific interactions between pathogens and the host, involving the early interaction with the innate system, which includes mechanical, chemical and cellular barriers. Mucociliary clearance is one of the first mechanical defences faced by any pathogen in the respiratory tract. Pathogens may be trapped in a blanket of mucus which covers the airways and is constantly propelled by cilia from the distal to proximal lung airways. The flow of urine in conjunction with its low pH prevents colonisation of the genitourinary tract, whereas peristalsis and the mucus lining of the gastrointestinal tract limit the attachment of bacteria to the gut epithelium. The presence of digestive enzymes, bile and the acid pH in the stomach further prevents pathogen colonisation of the gastrointestinal tract.

Once pathogens overcome these mechanical barriers, they face the challenge of chemical defences, chiefly the complement system, collectins and antimicrobial peptides. In the classical pathway of activation of the complement cascade, C1q recognises pathogen- or damage-associated patterns (such as IgG, IgM or CRP) on foreign or apoptotic cells, inducing the formation of the C3 convertase (C4b2b) (Holers 2014). In the lectin pathway, mannose-binding lectins and ficolins bind to carbohydrates leading to the activation of C4b2b, which subsequently activates C3 in its active fragments C3a and C3b (Holers 2014). The deposition of the latter on surfaces leads to the binding of factor B and conversion into the alternative pathway C3 convertase (C3bBb), which cleaves more C3 into C3b, thereby amplifying the complement response (Holers 2014). Opsonisation by C1q, C3b and its degradation products induces phagocytosis via a panel of complement receptors (Ricklin *et al.*^2016^). In addition, complement factors such as C3a and C5a are powerful chemoattractants guiding neutrophils, macrophages and monocytes to the sites of infection (Ricklin *et al.*^2016^). Complement also shapes inflammatory responses activated via pathogen recognition receptors (PRRs), and even dictates the differentiation of T cells, thereby acting as player and mediator in immune surveillance (Ricklin *et al.*^2010^).

Collectins are a family of proteins that include mannan-binding lectin (mannose-binding protein) and lung surfactant proteins (SPs), SP-A to D (Holmskov, Thiel and Jensenius 2003). These proteins share a common structure made of a C-terminal-located C-type lectin domain which is attached to a collagen-like region via an alpha-helical coiled-coil neck region (Holmskov, Thiel and Jensenius 2003). Collectins bind surface carbohydrates in pathogens leading to the opsonisation, agglutination or killing of the pathogen. Interestingly, lung SPs have also immunomodulatory roles by governing phagocytosis and controlling inflammation (Sano and Kuroki 2005; Kuroki, Takahashi and Nishitani 2007). Additional defences against bacterial infections include antimicrobial peptides and proteins, and cathelicidins produced by epithelial cells, neutrophils and macrophages in response to infection. The levels of these antimicrobials in the site of infection may reach hundreds of micrograms creating a harsh environment. Defensins and lysozyme have potent antibacterial activity against Gram-positive and -negative bacteria. The antibacterial action is based on electrostatic interaction with the anionic bacterial surface leading to membrane perturbations. LL-37/hCAP-18, the only cathelicidin found in humans, is also antimicrobial. Interestingly, defensins and cathelicidins have additional multiple roles influencing diverse processes such as cell proliferation and migration, immune modulation, wound healing, angiogenesis and the release of cytokines (Ganz 2003; Bowdish, Davidson and Hancock 2006).

Upon infection, the host activates a sophisticated program dedicated to clear the pathogen by activation of germ-line encoded receptors referred to as pathogen recognition receptors (PRRs). Data support the notion that there is a common host response associated to infection, the so-called ‘alarm signal’, mainly controlled by PRRs (Jenner and Young 2005; Lachmandas *et al.*^2016^; Li *et al.*^2016^; Martinez *et al.*^2017^). Elements of this antimicrobial programme are antimicrobial molecules, cytokines, chemokines and IFNs. Early production of type I IFN is required to limit initial viral replication. However, type I IFN-dependent responses can no longer be considered virus specific since a body of mainly recent data indicates that type I IFNs are also produced in response to bacteria. However, depending on the bacterial infection, type I IFNs exert seemingly opposing functions (Boxx and Cheng 2016; Kovarik *et al.*^2016^).

The most extensively studied mammalian (human and mouse) PRRs belong to the ‘Toll-like’ receptors (TLRs), the nucleotide-binding and oligomerisation domain-like receptors (NLRs) and the retinoic acid inducible gene I (RIG-I)-like receptor (RLR) families (Takeuchi and Akira 2010). Activation of all these PRRs converges on the activation of mitogen-activated protein kinases (MAPKs), and a limited set of transcriptional factors, mainly IRF3, IRF7 and NF-κB. These factors act cooperatively to activate the transcription of genes. Several members of the NLR protein family, NLRP1, NLRP3, NLRC4, may assemble into a multiprotein platform, known as inflammasome, to induce caspase-1 activation (Latz, Xiao and Stutz 2013; Guo, Callaway and Ting 2015). This protease is responsible for the cytosolic processing of pro-IL-1β and pro-IL-18 and for the secretion of their mature active forms. IL-1β and IL-18 exert crucial roles orchestrating immune responses to control infections. Activation of caspase-1 also triggers a form of cell death called ‘pyroptosis’. The role of pyroptosis as a *bona fide* cell-autonomous defence mechanism is still poorly understood, although recent evidence indicates that pyroptosis triggers pore-induced intracellular traps that capture bacteria and lead to their clearance by efferocytosis (Miao *et al.*^2010^; Jorgensen *et al.*^2016^). Other inflammatory caspases, caspase-11 in mouse and caspases4/5 in humans, detect cytosolic lipopolysaccharide (LPS) and trigger the activation of the so-called non-canonical inflammasome to produce IL1β and induce cell death (Hagar *et al.*^2013^; Shi *et al.*^2014^).

Several PRRs detect RNA (Schlee and Hartmann 2016). TLR3 and TLR7 detect double-stranded RNA in the endosome, whereas TLR7 and TLR8 sense single-stranded RNA. The helicases RIG-I and melanoma differentiation associated gene 5 (MDA5) also detect double-stranded RNA in the cytosol. Stimulation of these receptors results in the production of type I IFN, as well as the expression of IFN-stimulated genes. There is still limited knowledge on the possible contribution of these RNA-sensing receptors to bacterial defence.

Only in the past years, the molecular basis of cytosolic DNA sensing by the innate immune system has begun to be revealed (Paludan and Bowie 2013). Several DNA sensors were identified, and a new family of DNA sensors called AIM2-like receptors formed by, at least, IFI16, AIM2 and p202 (a negative regulator of AIM2), which are all PYHIN proteins, has been proposed. STING is the central adaptor for cytosolic DNA sensing directing TBK1 to activate IRF3 for DNA sensing pathways. The role of STING during bacterial diseases is controversial, ranging from protective to detrimental effects for the host (Marinho *et al.*^2017^).

## MODELS TO ASSESS *KLEBSIELLA* SPECIES INFECTION BIOLOGY

Several research models have been implemented to assess *Klebsiella* infection biology (Table 1). However, the vast majority of evidence on the interplay between *Klebsiella* species and the immune system has been obtained by infecting rodents, chiefly mice. Inbred mouse strains allow the study of genetically identical cohorts, whereas the development of methods for the creation of transgenic, knock-out and knock-in mice has provided powerful tools to investigate *Klebsiella* infection biology. Outcomes of infection vary with the mouse strain used, infection with 10^2^ CFUs leads to bacteremia and death within 72 h in CD-I, CBA/J and BALB/c mice while C57BL/6 mice are more resistant (Mehrad and Standiford 1999). The mouse model has been extensively used to investigate two clinical manifestations of *Klebsiella* infections: pneumonia and sepsis. Studies of *Klebsiella-*induced pneumonia in animal models date back to 1947 with induction of pneumonia in rats with intratracheal instillation of *K. pneumoniae* (Sale, Smith and Wood 1947; Sale and Wood 1947). The mouse pneumonia model recapitulates key features of *Klebsiella*-induced pneumonia in humans, namely the massive inflammation characterised by an influx of polymorphonuclear neutrophils, and oedema. The results obtained with this model have uncovered receptors and molecules implicated in host defence against the pathogen. Moreover, the mouse model has been useful to provide mechanistic evidence on why some health factors such as alcohol abuse, obesity, poor glycaemic control in diabetic patients and respiratory viral infection are associated with increased susceptibility to *Klebsiella* infections (Mancuso *et al.*^2002^; Happel *et al.*^2006^; Ballinger and Standiford 2010).

**Table 1.** Models to assess *K. pneumoniae* infection biology.

Infection model	Useful to assess	Advantages	Limitations
*Mus musculus*	Recapitulates many clinical aspects of *Klebsiella* infections.	Well-established model. Available knock-out and knock-in animals.	Costs. Differences between mice and human immune system.
*Dyctiostelium discoideium*	Recapitulates interactions with phagocytic cells.	Easy to handle. Available genetic tools, and bank of mutant strains.	Growth conditions (temperature and growth medium to assess virulence).
*Drosophila melanogaster*	Early interactions with ancient antibacterial mechanisms.	Easy to handle. Available genetic tools, and bank of mutant strains.	Growth conditions. Not clear how to translate findings in this model to model human disease.
*Caenorhabditis elegans*	Early interactions between a pathogen and a host.	Easy to handle. Available genetic tools, and bank of mutant strains.	Growth conditions. Not clear how to translate findings in this model to model human disease.
*G. mellonella*	Recapitulates interactions with innate immune system (effectors and phagocytes).	Easy to handle. Growth conditions (temperature). Good correlation with the mouse model in terms of assessing virulence.	Lack of genetic tools, and bank of mutant strains.
*Danio rerio*	Interactions with a complex immune system	Possibility of easily imaging infection. Available genetic tools, and bank of mutant strains.	Need for costly infrastructure. Not clear how to translate findings in this model to model human disease.

In the mouse model, *Klebsiella*-induced pneumonia is achieved either via intratracheal/endobronchial instillation or via intranasal inoculation, each of which has its limitations. Intratracheal/endobronchial instillation delivers the inoculum to the lower respiratory track bypassing the host barriers of the upper airways but modelling oropharyngeal aspiration. The intratracheal method of infection often results in a higher ratio of infecting organisms to local defences at the site of infection than those achieved with other inoculation routes. This results in an exuberant inflammatory response and tissue destruction already few hours after infection. We suggest that the intratracheal/endobronchial inoculation route should be the one choice to investigate the biology of *Klebsiella*-induced lung injury. However, to gain insights into *Klebsiella-*triggered respiratory infections, we favour the intranasal inoculation route because it captures the interaction between the pathogen and the defences of upper and lower respiratory track. Furthermore, it is a simple method of infection. Its major limitation is the variable deposition of microorganisms in the lungs which may lead to significant differences between infected mice.

Intratracheal, intraperitoneal and intravenous routes of infection are used to model *Klebsiella*-triggered sepsis, whereas intraperitoneal and orogastric routes of infection are used to investigate the virulence of *Klebsiella* strains inducing pyogenic liver abscess (Siu *et al.*^2012^). This syndrome was anecdotally reported in Taiwan in the 1980s, although now it seems to be spreading to countries outside Asia. Clinical evidence suggests that healthy adults carry the virulent strains in their intestines, and liver abscess occur when bacteria translocate across the intestinal epithelium (Siu *et al.*^2012^). Experiments done in mice provide initial evidence supporting this notion (Tu *et al.*^2009^); however, it should be noted that this infection model only demonstrates the ability of these strains to spread from the gut to other organs. Of note, there are no specific *Klebsiella* genetic features associated with these infections, suggesting that perhaps host factors might play a critical role in the outcome of this *Klebsiella*-triggered pyogenic liver abscess. Currently, there is no well-developed model to investigate the gut colonisation by *Klebsiella* species. Recently, Krapp and colleagues (Krapp *et al.*^2017^) have developed a subcutaneous model of infection to model *Klebsiella*-induced skin and soft tissue infections. These are rare clinical manifestations also associated with hypervirulent strains.

Although the mouse model has proven useful to illuminate *K. pneumoniae* infection biology, it is important to acknowledge its limitations. There exist significant differences between mice and humans in immune system development, activation and response to infection (Mizgerd and Skerrett 2008). For example, circulating neutrophil counts are lower in mice than in humans (Haley 2003), and mouse neutrophils lack defensins (Eisenhauer and Lehrer 1992). There are no murine homologs of several chemokines including IL8, although mice express other chemoattractant cytokines (Strieter *et al.*^1996^). Species differences also exist in the receptors sensing infections. There are 10 known functional TLRs in humans and 12 in mice (Takeuchi and Akira 2010); TLRs 1–9 are conserved in both species, although the tissue expression and transcriptional regulation also differ (Rehli 2002). There is general conservation between mouse and human TLR-controlled signalling pathways; however, there are notable differences in the use of signalling adaptors (Sun *et al.*^2016^). In addition, the ligand specificities and affinities of TLRs also differ in humans and mice. For example, human TLR4 exquisitely discriminates between lipid A structures, whereas murine TLR4 does not and, as result, there are differences in the inflammatory responses induced by lipid As with different structures (Hajjar *et al.*^2002^; Montminy *et al.*^2006^). These discrepancies and others (Mizgerd and Skerrett 2008) should be carefully considered in interpreting experiments to translate the findings to human disease.

More recently, other non-mammalian infection models have been tested to investigate *Klebsiella* pathogenesis to circumvent ethical and costs issues associated with animal research, and to potentially facilitate large-scale analysis of virulence. The social amoeba *Dictyostelium discoideum* is a phagocytic cell that can be used to screen potential roles of phagocytic immune cells such as neutrophils and macrophages (Dunn *et al.*^2018^). There is evidence demonstrating that *D. discoideum* is a valuable system for studying how pathogens evade fundamental processes of phagocytic cells (Dunn *et al.*^2018^). Benghezal and colleagues (Benghezal *et al.*^2006^) carried out a two-dimensional virulence array to identify *D. discoideum* genes implicated in host defence against *Klebsiella*, and *Klebsiella* genes require to survive in the attenuated *D. discoideum* host. *phg1* and *kil1,* the *D. discoideum* genes identified as essential to kill intracellular *Klebsiella*, have homologues in mammalian cells, although their potential contribution to the physiology of phagocytic cells has not been studied yet. The screen of bacterial mutants revealed that the surface polysaccharides expressed by *Klebsiella*, the capsule polysaccharide (CPS) and the LPS play a crucial role in the interaction with *D. discoideum* (Benghezal *et al.*^2006^). Additional studies have shown that, in addition to the CPS and the LPS O-polysaccharide and core, the outer membrane proteins (OMP) OmpA and OmpK36, and the lipid A decorations with aminoarabinose and palmitate are also necessary to avoid predation by *D. discoideum* (March *et al.*^2013^). Interestingly, these factors are also required to limit phagocytosis by mouse alveolar macrophages (March *et al.*^2013^), suggesting that *K. pneumoniae* exploits the same factors to interact with social amoeba and macrophages. *Dictyostelium discoideum* has also proven to be useful to model the interaction between *Klebsiella* and human neutrophils (Pan *et al.*^2011^), where CPS and LPS O-polysaccharide being also the important factors governing the interaction of *Klebsiella* with neutrophils. Further reinforcing the importance of *Klebsiella* surface polysaccharide on the interaction with phagocytes, *D. discoideum* specifically senses *Klebsiella* CPS to activate a predation programme (Lima *et al.*^2014^).

The nematode *Caenorhabditis elegans* and *Drosopila melanogaster* have also been used to identify host pathways implicated in host defence against *Klebsiella*. In *C. elegans*, PI3K-AKT/mTOR and the MAPK p38 are required for host protection against *K. pneumonia* (Kamaladevi and Balamurugan 2015, 2017), whereas Phg1, important in the *Klebsiella-D. discoideum* interplay, is also essential to resist *K. pneumoniae* infection by *D. melanogaster* (Benghezal *et al.*^2006^). Whether these pathways play any role in mammalian defence against *K. pneumoniae* is currently unknown.

A common limitation of these models is that the optimal temperature for maintaining them is 28°C, whereas the optimum temperature for *K. pneumoniae* is 37°C. Since virulence gene expression is frequently regulated by temperature, it is likely that temperature requirements may affect the interaction of *K. pneumonaie* strains causing human infections with these hosts. Nonetheless, it is important to note that the impact of environmental cues on the regulation of *Klebsiella* virulence factors is poorly understood. The larvae of the wax moth *Galleria mellonella* is emerging as a suitable model to study the virulence of many human pathogens because, among other advantages, it grows at 37°C (Table 1) (Glavis-Bloom, Muhammed and Mylonakis 2012; Tsai, Loh and Proft 2016). *G. mellonella* defence against bacterial infections consists of cellular and humoral immunity (Glavis-Bloom, Muhammed and Mylonakis 2012; Browne, Heelan and Kavanagh 2013; Wojda 2017). The cellular response is mediated by phagocytic cells, termed hemocytes, found within the haemolymph. Hemocytes govern the clotting reaction to trap pathogens, and the melanisation response consisting of the deposition of melanin to encapsulate pathogens at the site of infection followed by the coagulation of hemolymph. Melanisation can be considered analogous to abscess formation in mammalian infections. The humoral response is orchestrated by soluble effector molecules that immobilise or kill the pathogen and includes complement-like proteins, and antimicrobial peptides. The suitability of *G. mellonella* as a model to investigate *K. pneumoniae* pathogenesis has only been recently demonstrated (Insua *et al.*^2013^). This infection model discriminates the pathogenic potential of *K. pneumoniae* strains (Insua *et al.*^2013^; Wand *et al.*^2013^), and there is a strong correlation with the virulence previously determined in the mouse pneumonia model (Insua *et al.*^2013^). Furthermore, *K. pneumoniae* infection of *G. mellonella* results in responses similar to those reported in the mouse pneumonia model including cell death-associated with bacterial replication, inhibition of phagocytosis and attenuation of host defence responses, chiefly the production of antimicrobial peptides (Insua *et al.*^2013^). Interestingly, virulence factors necessary in the mouse pneumonia model, CPS, LPS and OMPs, are also required for *K. pneumoniae* survival in *G. mellonella* (Insua *et al.*^2013^). The fact that all attenuated *Klebsiella* mutants activate *G. mellonella* defensive responses (Insua *et al.*^2013^) supports the notion that prevention of host responses is an important feature of *K. pneumoniae* pathogenesis. Despite the clear utility of *G. mellonella* as a surrogate host to assess infections with *K. pneumoniae*, it is important to note that the model only reflects early features of the interaction between the pathogen and ancient innate immune mechanisms of defence. These mechanisms are indeed conserved in evolution; however, the evolutionary distance between insects, mice and humans makes that many host-specific phenomena are likely to exist.

The larvae of *Danio rerio* (zebrafish) is another non-mammalian infection model receiving increasing attention because it is genetically tractable, optically accessible and present a fully functional innate immune system with macrophages and neutrophils that mimic their mammalian counterparts (Torraca and Mostowy 2018). Although a wide variety of pathogenic bacteria have been already investigated using zebrafish, only recently it has been assessed whether zebrafish larvae are a suitable model to investigate *K. pneumoniae* virulence (Cheepurupalli *et al.*^2017^; Marcoleta *et al.*^2018^). These studies report the optimisation of the model to investigate *K. pneumoniae* pathogenesis. Injection of larvae seems to be the most reliable inoculation method to ensure consistent colonisation of the gastrointestinal tract (Cheepurupalli *et al.*^2017^). Further studies are warranted to validate whether the model is useful to identify *Klebsiella* virulence factors and to uncover features of the interaction between *K. pneumoniae* and the immune system.

## CONTRIBUTION OF HOST SIGNALLING IN DEFENCE AGAINST *K. PNEUMONIAE* INFECTIONS

**Figure 1.**
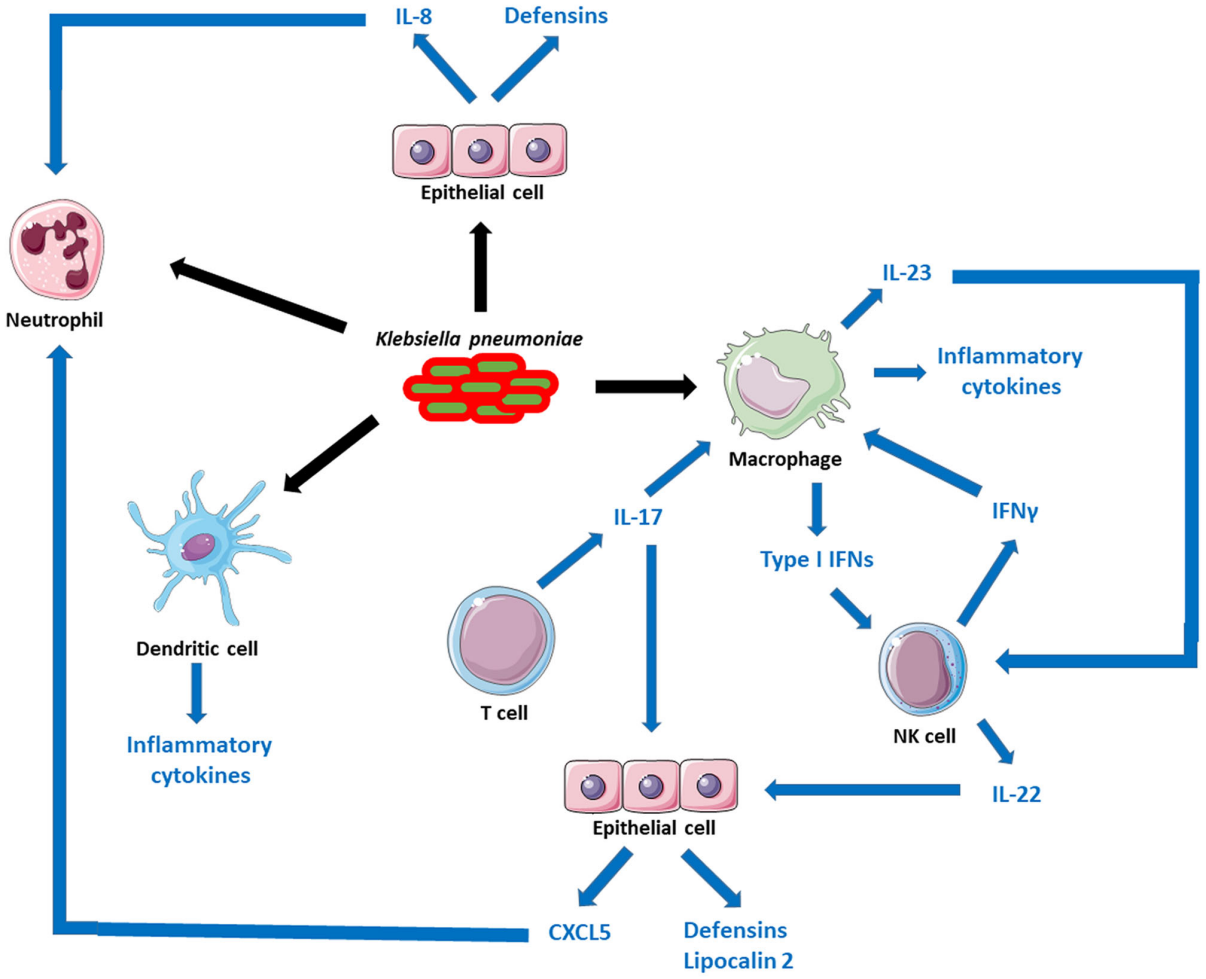
Mechanisms of innate immunity to *K. pneumoniae* infections. The figure depicts the cells implicated in containing *K. pneumoniae* infection. There is conclusive evidence demonstrating the interaction of *K. pneumoniae* with neutrophils, macrophages (and monocytes [not shown]), dendritic cells and epithelial cells. These interactions are marked with black arrows. The interaction with different subset of T cells, NK cells and other lymphocytes has not been investigated yet, although these cells participate in bacterial clearance. The network of connections between cells, and the role played by different cytokines activating host responses are depicted with blue arrows.

The published evidence during more than 20 years clearly establishes that pro-inflammatory signalling is crucial to *K. pneumoniae* clearance (Fig. 1). One of the first conclusive piece of evidence showed that mice deficient in the TNFα receptors (TNFR1) are susceptible to *K. pneumoniae* pneumonia (Laichalk *et al.*^1996^, ^1998^; Moore *et al.*^2005^). Subsequent studies reported that mice lacking the chemokines CXCL15 (Chen *et al.*^2001^) and CCL3 (Lindell *et al.*^2001^), and unable to synthesise leukotrienes (Bailie *et al.*^1996^; Mancuso *et al.*^1998^) and nitric oxide (Tsai *et al.*^1997^) are also exquisitely susceptible to *Klebsiella* pneumonia. All these are markers associated with human pneumonia and are part of the common host response to infections (Jenner and Young 2005; Lachmandas *et al.*^2016^; Li *et al.*^2016^; Martinez *et al.*^2017^). In turn, strategies to boost pro-inflammatory signalling in the airways have proven to be successful to limit *K. pneumoniae* infections. Intrapulmonary expression of CCL3 (Zeng *et al.*^2003^), and KC (Tsai *et al.*^1998^; Cai *et al.*^2010^) leads to improved clearance of *K. pneumoniae*. Intratracheal instillation of CpG increases the production of TNFα, and Th1 cytokines, including IL-12, IFNγ and the IFNγ-dependent ELR-CXC chemokines (Deng *et al.*2004a) in a TLR9-dependent manner (Bhan *et al.*^2007^), enhancing bacterial clearance. Cyclic di-GMP, a molecule sensed by STING (Burdette *et al.*^2011^), also triggers a vigorous expression of chemokines and Th1 cytokines (Karaolis *et al.*^2007^). These treatments also result in the increased recruitment of neutrophils, αβ and γδ T cells, and activated NK cells to the site of infection, suggesting that these cells are crucial in host defence against *K. pneumoniae*. Indeed, γδ T cells and NK cells play a pivotal role in the resolution of *Klebsiella* infections by controlling early production of pro-inflammatory cytokines (Moore *et al.*^2000^; Xu *et al.*^2014^).

The role of different cytokines in host defence against *K. pneumoniae* has been also investigated (Fig. 1). Early studies demonstrated the importance of IFNγ and IFNγ-dependent cytokines to control the progression of *Klebsiella*-induced pneumonia (Yoshida *et al*. 2001; Moore *et al.*^2002^; Zeng *et al.*2005a,b). IL23 drives the production of IFNγ and IL17 (Happel *et al.*^2003^, ^2005^); however, the fact that IL17 administration restores bacterial control in mice deficient on IL23 production indicates an independent role for the IL17-governed axis on host defence against *K. pneumoniae* (Happel *et al.*^2005^). Adding further evidence to this notion, IL17 signalling is critical for the induction of Th1 responses, neutrophil recruitment and local control of pulmonary infection (Ye *et al.*2001a,b). IL17 signalling is also augmented via IL12 production through IFNγ (Happel *et al.*^2005^). The contribution of IL12 signalling to control *Klebsiella* pneumonia is exemplified by the fact that *STAT4^−^^/^^−^* knock-out mice displayed impaired production of IFNγ and Th1 cytokines and greater bacterial burden compared to wild-type infected mice (Deng *et al.*2004b). STAT4 is a critical transcriptional factor in the IL12 signalling pathway (Bacon *et al.*^1995^). IL22, produced in an IL23-dependent manner, is also important in host defence against *K. pneumoniae* (Aujla *et al.*^2008^; Zheng *et al.*^2016^). Administration of an anti-IL22 blocking antibody results in higher bacterial loads in the lungs and dissemination of bacteria to spleen (Aujla *et al.*^2008^), whereas therapeutic administration of IL22 attenuates *Klebsiella*-triggered peritonitis (Zheng *et al.*^2016^). IL22 regulates the levels of IL6 and CCL3 upon *Klebsiella* infection, and its role is predominant over IL17 in regulating these cytokines (Aujla *et al.*^2008^). The synergistic effect of both cytokines governing host defences against *Klebsiella* is marked by the fact that neutralisation of IL22 in *Il17a^−/−^* mice results in greater bacterial growth in the lung and significantly more bacterial dissemination to the spleen than in those observed in infected *Il17a^−/−^* (Aujla *et al.*^2008^).

Collectively, the summarised evidence strongly suggests that the IL23/IL17 and IL12/IFNγ axes are essential for the generation of an effective innate immune response in the lungs against *K. pneumoniae*. However, two questions require additional investigations: Which are the cell(s) responsible for the production of these cytokines? and Which are the main innate defence mechanisms (humoral and cellular) activated by these cytokine networks responsible for the clearance of *K. pneumoniae*? To set the framework for future studies, we next discuss the available evidence. Initial data suggest that γδ T and NK cells could be the major source of IL17 and IL22, respectively, in *Klebsiella*-infected mice (Xu *et al.*^2014^; Murakami *et al.*^2016^), whereas alveolar macrophages could be the initial source of IL23 (Happel *et al.*^2005^). Only recently, it has been also suggested that type 3 innate lymphocytes could be another source of IL17 *in vivo* (Xiong *et al.*^2016^). Altogether, there is need to dissect the source of IL17 during *K. pneumoniae* infections. Dendritic cells have been shown to orchestrate the production of IL12, IL23 and IL17 *in vivo* (Bhan *et al.*^2007^), although the specific singular role of dendritic cells in *Klebsiella* infections and the connection with other immune cells have not yet been fully defined. There is evidence showing that NK cells are the source of IFNγ in *Klebsiella*-infected mice (Van Elssen *et al.*^2010^; Ivin *et al.*^2017^), although it cannot be ruled out that other cell types such as CD4 and CD8 T cells might contribute as well. Alveolar macrophages and recruited inflammatory monocytes are considered the main cellular target for IFNγ and IL17, respectively (Xiong *et al.*^2015^, ^2016^; Ivin *et al.*^2017^). These cytokines enhance the microbicidal activity of alveolar macrophages and inflammatory monocytes by increasing phagocytosis and facilitating bacterial killing (Xiong *et al.*^2015^, ^2016^; Ivin *et al.*^2017^). Whether IFNγ and IL17 trigger other antimicrobial activity on these cells remains to be investigated. IL17 and IL22 also aid in the clearance of *Klebsiella* by regulating the antimicrobial activity of the lung epithelium (Aujla *et al.*^2008^; Chen *et al.*^2016^). Both cytokines activate antimicrobial programmes in epithelial cells having in common the production of CXCL5 and lipocalin 2 (Aujla *et al.*^2008^; Chen *et al.*^2016^). Ablation of this programme results in higher bacterial loads in the lungs of infected animals. CXCL5 participates in the recruitment of neutrophils to the site of infection (Chen *et al.*^2016^), whereas lipocalin 2 inhibits the growth of some strains of *Klebsiella in vitro* and *in vivo* by preventing bacterial iron acquisition (Bachman, Miller and Weiser 2009; Chan *et al.*^2009^; Bachman *et al.*^2012^). Additionally, lipocalin 2 may also promote the induction of pro-inflammatory responses, which facilitates the recruitment of neutrophils like CXCL5 does. However, it is important to be aware that there are conflicting reports on the role of neutrophils *in vivo* to clear *Klebsiella* infections (Greenberger *et al.*^1996^; Broug-Holub *et al.*^1997^; Xiong *et al.*^2015^). Therefore, the recruitment of neutrophils to the lungs of *Klebsiella*-infected mice cannot be rigorously taken as conclusive evidence of these cells being a major player in host defence against the pathogen. *In vivo* mechanistic studies involving selective depletion of neutrophils together with *ex vivo* experiments testing isolated mouse neutrophils should be the norm in these type of studies.

Only recently, the role of type I IFN and type I IFN-governed signalling in host defence against *Klebsiella* infections has been investigated (Fig. 1). Alveolar macrophages are one of the sources of type I IFN *in vivo*, and *in vitro* experiments revealed that *K. pneumoniae* activates a TLR4-TRAM-TRIF-IRF3 signalling pathway to induce type I IFN- and type I IFN-dependent genes (Ivin *et al.*^2017^). To assess the importance of type I IFN signalling in host defence, researchers infected mice lacking type I IFN 1 receptor-deficient (*Ifnar1^−^^/^^−^*) mice (Ivin *et al.*^2017^). Ifnar1 is one of the subunits of the type I IFN receptor which mediates type I IFN responses in innate and acquired immunity to infection. *Ifnar1^−^^/^^−^* are exquisitely sensitive to *Klebsiella* infection exhibiting a markedly decreased survival, higher bacterial lung burden, increased dissemination to spleen and liver and severe bronchopneumonia (Ivin *et al.*^2017^). The lack of type I IFN signalling results in defect in the production of IFNγ, IL-12 and CXCL10 in *K. pneumoniae*-infected lungs, but it has no impact on the number of alveolar macrophages, inflammatory monocytes and neutrophils recruited to the site of infection (Ivin *et al.*^2017^). In contrast, the number of NK cells was lower in the lungs of *Ifnar1^−^^/^^−^*-infected mice than in the wild-type ones (Ivin *et al.*^2017^). This is consistent with the reduced levels of the NK chemoattractant chemokine CXCL10 in the lungs of *Ifnar1^−^^/^^−^*-infected mice (Ivin *et al.*^2017^). Type I IFN signalling is crucial for NK cells to produce IFNγ, which is required for enhancing the bactericidal action and the production of the NK cell response-amplifying IL-12 and CXCL10 by alveolar macrophages (Ivin *et al.*^2017^). Remarkably, type I IFN signalling is dispensable in myeloid cells including alveolar macrophages, monocytes and neutrophils for host defence and IFNγ activation (Ivin *et al.*^2017^), uncovering a hitherto unknown crosstalk between alveolar macrophages and NK cells based on type I IFN and IFNγ in *Klebsiella* infection.

Few studies have addressed the contribution of PRR-governed signalling to control *Klebsiella* infections. As with other bacterial infections, TLR4 signalling plays a prominent role in antibacterial defence against *Klebsiella* infection (Branger *et al.*^2004^; Wieland *et al.*^2011^; Standiford *et al.*^2012^). *TLR4^−^^/^^−^* mice show reduced survival upon infection with increased bacterial loads in lungs and bronchopneumonia (Branger *et al.*^2004^; Wieland *et al.*^2011^; Standiford *et al.*^2012^). The lack of TLR4 signalling is associated with a decrease in the levels of IL17 and IL23 in the lungs of infected *TLR4^−^^/^^−^* mice (Happel *et al.*^2003^), which may explain the susceptibility of these mice to *Klebsiella* infection. It remains an open question which cells are more affected by the lack of TLR4 signalling. Initial observations suggest that TLR4 signalling is indispensable in cells of myeloid origin for the clearance of *Klebsiella* (Wieland *et al.*^2011^); however, it cannot be ruled out that other cell types may require TLR4 signalling to aid in the elimination of *Klebsiella* infections. Supporting this notion, TLR4 signalling is required to protect the lung epithelium from *Klebsiella*-induced pathophysiology (Standiford *et al.*^2012^). TLR9-controlled signalling is also required for protective immunity against *Klebsiella*-induced pneumonia (Bhan *et al.*^2007^, ^2010^). Mice deficient in TLR9 fail to generate an effective Th1 cytokine response, resulting in increased bacterial loads in the lungs and dissemination to other organs (Bhan *et al.*^2007^). *TLR9^−^^/^^−^*-infected mice present no major defects on the accumulation of immune cells except on the influx and maturation of conventional dendritic cells (Bhan *et al.*^2007^). This reduced accumulation and activation of dendritic cells explains the impaired bacterial clearance because adaptive transfer of dendritic cells from wild-type mice reconstitutes the protective immunity in *TLR9^−^^/^^−^* mice (Bhan *et al.*^2007^). Since TLR9 is located in endosomes and it recognises DNA oligonucleotides with unmethylated CpG base pairs (Hemmi *et al.*^2000^), it is intriguing to consider how *Klebsiella* infection may lead to the activation of this intracellular receptor. Data obtained in other infection models indicate that TLR9 can be activated by bacterial and host DNA released into the airways during pneumonia (van der Meer *et al.*^2016^), as well as by intracellular bacteria and DNA of mitochondrial origin released to the cytosol upon infection (Zhang *et al.*^2010^; Arpaia *et al.*^2011^). Future studies should address this knowledge gap. TLR2 signalling has a dual role in host defence against *Klebsiella* (Wieland *et al.*^2011^). In the early phase of infection, TLR2 signalling has an anti-inflammatory role (Wieland *et al.*^2011^), perhaps to prevent a detrimental overwhelming inflammation as a result of the activation of other PRRs. Similar observation has been done in *Acinetobacter baumannii*-triggered pneumonia (Knapp *et al.*^2006^), suggesting that the dampening function of TLR2 during pneumonia is not bacterial species-specific. In the later stage of infection, TLR2 contributes to antibacterial defence (Wieland *et al.*^2011^). Interestingly, cooperative roles of TLR4 and TLR2 signalling are involved in controlling *Klebsiella* infection because *TLR4^−^^/^^−^xTLR2^−^^/^^−^* mice are more susceptible to the infection than each of the single knock-out mice (Wieland *et al.*^2011^).

The role of TLR signalling during *K. pneumoniae* infection has been further probed by demonstrating the contribution of TLR adaptors in host defence. MyD88 is the universal adaptor for all TLRs except TLR3 (O’Neill and Bowie *et al*. 2007), and it has been shown to be important for pulmonary host defence against several respiratory pathogens (Baral *et al.*^2014^). TRIF is the sole adaptor for TLR3 and also contributes to TLR4 signalling (O’Neill and Bowie *et al.*^2007^). Infections of *MyD88^−^^/^^−^* and *TRIF^−^^/^^−^* mice demonstrated that both adaptors are required to restrict *K. pneumoniae* growth in the lungs (Cai *et al.*^2009^; van Lieshout *et al.*^2012^). MyD88-dependent protection during *Klebsiella* pneumonia is mediated by both hematopoietic and resident cells excluding endothelial cells, whereas TRIF-mediated protection is driven by hematopoietic cells (van Lieshout *et al.*^2012^, ^2014^; Anas *et al.*^2017^). MyD88 and TRIF deficiencies limit the production of Th1 cytokines and the activation of signalling pathways controlling host defence mechanisms (Cai *et al.*^2009^). Interestingly, the characteristic bronchopneumonia of *Klebsiella* infections is virtually absent in infected *MyD88^−^^/^^−^* mice and significantly reduced in *TRIF^−^^/^^−^* mice despite high bacterial loads in both mice (Cai *et al.*^2009^). This evidence indicates that the histopathological changes associated with *Klebsiella* infection are dependent on the host inflammatory response to the infection. TIRAP/MAL is another adaptor linking MyD88 to the activated TLR2 and TLR4 receptors (O’Neill and Bowie *et al.*^2007^). In this context, it is not surprising that *MAL^−^^/^^−^* mice have substantial mortality, higher bacterial burden in the lungs, enhanced bacterial dissemination, attenuated production of Th1 cytokines and no lung histopathology following *K. pneumoniae* infection (Jeyaseelan *et al.*^2006^). At present, the mechanisms underlying MyD88-MAL-mediated defence against *Klebsiella* are ill-defined. However, it is important to note here that MyD88-MAL are also required for the activation of other signalling pathways such as those governed by IL1β and IFNγ (Cohen 2014; Ni Cheallaigh *et al.*^2016^). Therefore, MyD88-MAL-dependent protective immunity is most likely also mediated by IL1β- and IFNγ-governed host antibacterial responses. Likewise IFNγ-deficient mice, *IL1R^−^^/^^−^* mice are exquisitely susceptible to *Klebsiella* infection demonstrating the importance of IL1β-controlled responses for host survival and bacterial clearance (Cai *et al.*^2012^). On the other hand, the impaired antibacterial defence of *TRIF^−^^/-^*mice is associated with the lack of IFNγ in the lungs of infected mice (van Lieshout *et al.*^2015^). The fact that TRIF is required for type I IFN production following *Klebsiella* infection (Ivin *et al.*^2017^) suggests that the impairment of IFNγ production in *TRIF^−^^/–^*mice is secondary to the deficient type I IFN production in these mice.

A small number of studies have investigated the contribution of NLR signalling to defence against *Klebsiella. NLRP3^−^^/^^−^* mice demonstrate an increase in mortality following *Klebsiella* infection albeit the protective role of NLRP3 is not as important as those of TLR4 and TLR2, and any of the TLR adaptors (Willingham *et al.*^2009^). *In vitro* experiments confirmed the contribution of NLRP3 to caspase-1 activation and IL1β release following *Klebsiella* infection (Willingham *et al.*^2009^). In good agreement, *Klebsiella*-induced IL1β is reduced in *NLRP3^−^^/^^−^* mice (Willingham *et al.*^2009^). Recent evidence supports that the CPS and the LPS are the *Klebsiella* components responsible for priming NLRP3, whereas *Klebsiella*-triggered ROS may be responsible for the activation (Hua *et al.*^2015^). The NLRC4 inflammasome also contributes to *Klebsiella*-triggered IL1β production *in vitro* and *in vivo* (Cai *et al.*^2012^). However, there are contradictory results on the importance of NLRC4 to confer protection against *Klebsiella* infection (Willingham *et al.*^2009^; Cai *et al.*^2012^). The main apparent difference between studies is the different bacterial inoculum, with the study using an inoculum closer to the LD_50_ showing a contribution of NLRC4 on host immunity against *Klebsiella* (Cai *et al.*^2012^). Nonetheless, an open question is the identification of the *Klebsiella* component(s) inducing the activation of NLRC4. This receptor senses bacterial flagellin and the type III secretion system apparatus (Zhao *et al.*^2011^; Duncan and Canna 2018). Notably, *Klebsiella* is not flagellated and *in silico* analysis of more than 700 genomes confirms that this pathogen does not encode any type III secretion system. It is then tempting to speculate that any of the secretion systems encoded by *Klebsiella*, including the type II and type VI secretion systems, might be sensed by NLRC4. Intriguingly, *Klebsiella*-induced pyroptosis requires NLRP3 but not NLRC4 (Willingham *et al.*^2009^; Cai *et al.*^2012^). Whether pyroptosis is one of the *bona fide* host defence mechanisms against *Klebsiella* infection is yet unknown. In fact, the specific importance of pyroptosis in host immunity remains a challenging question. Initial evidence shows that NLRC4-dependent pyroptosis mediates the clearance of the intracellular pathogen *Salmonella typhimurium* by generating a structure that entraps the previously intracellular bacteria and drives their elimination by containing the bacteria and elaborating signals that promote efferocytosis (Miao *et al.*^2010^; Jorgensen *et al.*^2016^). However, it is unlikely that this mechanism operates in the context of *Klebsiella* infections because, as discussed before, the lack of NLRC4 does not affect *Klebsiella*-triggered pyroptosis.

## MICROBIOME PROTECTION AGAINST *KLEBSIELLA* INFECTIONS

There is a wealth of evidence demonstrating the role of the intestinal microbiota to prevent infection by pathogenic bacteria. This is achieved by interactions within the microbial community and by shaping the tissue immune responses to limit infection (McKenney and Pamer 2015). Surprisingly, there is virtually no data on the impact of the gut microbiome on *Klebsiella* gut colonisation and/or orogastric infection. This knowledge gap is particularly relevant considering the recent clinical evidence demonstrating that gastrointestinal carriage is a major reservoir of *K. pneumoniae* infections in the healthcare environment (Martin *et al.*^2016^; Gorrie *et al.*^2017^). On the other hand, the gut microbiota has been shown to protect against *Klebsiella* pneumonia. Experiments infecting germfree mice revealed that these animals are susceptible to *Klebsiella* in an IL-10–dependent manner (Fagundes *et al.*^2012^). In germfree mice, IL-10 in the lungs restrains pro-inflammatory mediator production and favours *Klebsiella* growth and dissemination (Fagundes *et al.*^2012^). Neutralisation of IL10, or transient TLR4 activation with LPS, restores germfree mice resistance to *K. pneumoniae* infection (Fagundes *et al.*^2012^). Subsequent studies provided compelling evidence on the members of the gut microbiota that drive protection against *Klebsiella* infection and the signalling pathways responsible for this microbiota-controlled immune protection. A consortium of bacterial species common to the rodent and human intestinal microbiota formed by *Lactobacillus reuteri, Enterococcus faecalis, Lactobacillus crispatus* and *Clostridium orbiscindens* induces potent NOD2 activation to trigger IL17-GM-CSF in the lung which, in turn, stimulates pathogen killing and clearance by alveolar macrophages through MAPK extracellular signal-regulated kinase signalling (Clarke 2014; Brown, Sequeira and Clarke 2017). The source of IL17 in the lung and how the microbiota governs the production of this cytokine remain open questions. Nevertheless, these findings further highlight the critical role of IL17 in host defence against *Klebsiella*, and the crucial role played by alveolar macrophages promoting antibacterial defence in the lung. Although the contribution of the upper airway microbiota, either permanent resident or aspirated from the oropharynx, to limit respiratory infections has not been demonstrated yet, it is notable that intranasal inoculation of bacteria colonising the upper airway of humans and mice (*Lactobacillus crispatus, Staphylococcus aureus, S. epidermidis*) enhances lung immunity against *Klebsiella* by the same IL17-GM-CSF-alveolar macrophage axis (Brown, Sequeira and Clarke 2017). Collectively, this evidence demonstrates the facility of the lung immune system to integrate microbial signals from different mucosal sites to launch antibacterial defence mechanisms.

## *KLEBSIELLA* EVASION STRATEGIES OF HOST DEFENCES

The widely held belief is that *K. pneumoniae* is a stealth pathogen, which fails to stimulate innate immune responses (Paczosa and Mecsas 2016). Essentially, *Klebsiella* shields its pathogen-associated molecular patterns from detection by PRRs and soluble effectors of the immune system, and avoids the interaction with hematopoietic and non-hematopoietic cells to prevent the activation of host antimicrobial responses (Table 2). However, there is now enough evidence demonstrating that *Klebsiella* also actively subverts host defences (Table 2). We will review both immune evasion strategies in the context of the interaction of *Klebsiella* with different effectors of the immune system.

**Table 2.** Immune evasion strategies of *K. pneumoniae*.

Immune evasion strategies	Mechanism	Bacterial factor	References
(i) Stealth pathogen			
Preventing the antimicrobial action of soluble innate immune effectors			
Preventing complement bactericidal effect, and opsonisation	Limiting C3b deposition	CPS, LPS O-polysaccharide	Merino *et al.*^1992^; Alvarez *et al.*^2000^; de Astorza *et al.*^2004^
Limiting antimicrobial activity of collectins	Blunting interaction with SP-A and SP-D	CPS	Kabha *et al.*^1997^; Ofek *et al.*^2001^; Kostina *et al.*^2005^
Counteracting bactericidal action CAMPs and polymyxins	Limiting the interaction with the bacterial surface. Efllux of CAMPs.	CPS, LPS lipid A decorations, AcrAB	Campos *et al.*^2004^; Llobet *et al.*^2011^; Kidd *et al.*^2017^; Mills *et al.*^2017^; Padilla *et al.*^2010^
Attenuating the interaction with immune cells			
Attenuating engulfment by epithelial cells		CPS	Cortes *et al.*^2002^; Regueiro *et al.*^2006^
Avoiding phagocytosis by neutrophils		CPS, OmpK36	Regueiro *et al.*^2006^; Pan *et al.*^2011^
Avoiding phagocytosis by macrophages		CPS, LPS lipid A decorations, OmpA, OmpK36	March *et al.*^2013^
Limiting the activation of PPRs	Limiting the recognition of LPS by TLR4	LPS lipid A 2-hydroxylation	Llobet *et al.*^2015^
(ii) Subversion host defences			
Attenuating cell-intrinsic immunity			
Controlling maturation dendritic cells		CPS, LPS O-polysaccharide	Evrard *et al.*^2010^
Manipulation phagosome maturation	Activation PI3K-AKT-Rab14 axis	Unknown	Cano *et al.*^2015^
Controlling cell death	Cytotoxicity in epithelial cells. Triggering apoptosis in macrophages.	CPS Unknown	Cano *et al.*^2009^; Leone *et al.*^2016^
Abrogating TLR-controlled inflammatory responses:			
Abolishing TLR signalling		CPS, LPS O-polysaccharide, OmpA, T2SS	March *et al.*^2011^; Frank *et al.*^2013^; Tomas *et al.*^2015^
Blunting NF-κB signalling	Upregulation deubiquitinase CYLD by targeting NOD1 and EGFR.	CPS, and other unknown factor(s)	Regueiro *et al.*^2011^; Frank *et al.*^2013^
Blunting MAPKs	Upregulation MAPKs phosphatase MKP-1 via NOD1 activation.	Unknown	Regueiro *et al.*^2011^
Manipulating mucosal immunity	Induction of IL10.	Unknown	Greenberger *et al.*^1995^; Yoshida *et al.*^2001^
Counteracting nutritional immunity	Secretion of several siderophores	Yersiniabactin, salmochelin, aerobactin	Lawlor, O’connor and Miller 2007; Bachman *et al.*^2011^; Russo *et al.*^2011^

### Counteracting soluble effectors of the immune system

Early research focused on investigating the interplay between complement and *K. pneumoniae*. The OMPs and LPS of *K. pneumoniae* are known to activate the classical pathway (Alberti *et al.*1996a,b). OmpK36 and OmpK35, homologues to OmpF and OmpC, respectively, and two of the most abundant porins in the outer membrane of *K. pneumoniae*, bind Cq1 in an antibody-independent manner triggering complement activation (Alberti *et al.*^1993^, 1996a). *K. pneumoniae* LPS without O-polysaccharide also activates the classical pathway, although less efficiently than the OMPs (Alberti *et al.*1996b). C3b deposition on the bacterial surface upon complement activation results in increased internalisation of *Klebsiella* by human lung epithelial cells promoting bacterial clearance (de Astorza *et al.*^2004^), as well as opsonophagocytosis by neutrophils and macrophages (Domenico *et al.*^1994^; Salo *et al.*^1995^; Regueiro *et al.*^2006^). Not surprisingly, the main complement evasion strategy of *Klebsiella* is based on preventing C3b deposition by exploiting *Klebsiella* surface polysaccharides. Whether *K. pneumoniae* may exploit other complement evasion strategies, such as targeting factor H, has not been described yet. The CPS is the main factor protecting *Klebsiella* from complement; *cps* mutants are susceptible to the bactericidal action of complement and show increased deposition of C3b on the surface (Merino *et al.*^1992^; Alvarez *et al.*^2000^). The protection conferred by CPS is more dependent on the thickness of the polysaccharide than the chemical composition of the polysaccharide (de Astorza *et al.*^2004^), although CPS containing manno(rhamno)biose may activate the lectin complement pathway (Sahly, Keisari and Ofek 2009). The LPS O-polysaccharide also protects *Klebsiella* from complement by limiting the deposition of C3b on the bacterial surface (Merino *et al.*^1992^). In those strains lacking the LPS O-polysaccharide, the CPS is then the main factor protecting *Klebsiella* from the bactericidal action of complement (Alvarez *et al.*^2000^).

The CPS also confers protection against lung collectins SP-A and SP-D, components of the lung surfactant, by preventing the binding of the collectins to the LPS (Kabha *et al.*^1997^; Ofek *et al.*^2001^; Kostina *et al.*^2005^). The binding of both collectins to the bacterial surface triggers bacterial agglutination and facilitates phagocytosis by macrophages (Kabha *et al.*^1997^; Ofek *et al.*^2001^; McCormack and Whitsett 2002). Interestingly, pulmonary surfactant challenge shapes the transcriptome of *K. pneumoniae*, inducing a programme strongly associated with virulence in the pneumonia mouse model (Willsey *et al.*^2018^). The CPS is one of the systems induced by pulmonary surfactant, further emphasising the importance of this polysaccharide to protect *Klebsiella* against collectins.

Like many other bacterial pathogens, *K. pneumoniae* has developed strategies to counteract host cationic antimicrobial peptides (CAMPs), chiefly defensins. Importantly, CAMPs and antibiotics such as quinolones and polymyxins share the same initial target in the outer membrane of Gram-negative bacteria (Nikaido 2003). Therefore, there is a relationship between resistance to CAMPs and polymyxins (Campos *et al.*^2004^; Campos, Morey and Bengoechea 2006; Nizet 2006; Llobet *et al.*^2011^; Kidd *et al.*^2017^). To counteract the bactericidal action of CAMPs and polymyxins, *K. pneumoniae* exploits the versatility of the CPS and the LPS. CPS limits the interaction of CAMPs and polymyxins with *Klebsiella* surface, and, in fact, there is a correlation between the amount of CPS expressed by a given strain and the resistance to polymyxin B (Campos *et al.*^2004^). Furthermore, free CPS, which may be released from the bacterial surface by CAMPs and polymyxins, binds CAMPs, neutralising their bactericidal effect (Llobet, Tomas and Bengoechea 2008). Therefore, the CPS acts as a bacterial decoy for CAMPs. Notably, this trait is shared by anionic CPS expressed by *Pseudomonas aeruginosa* and *Streptococcus pneumoniae* (Llobet, Tomas and Bengoechea 2008), strongly suggesting that trapping CAMPs is a general feature of anionic CPS.

*K. pneumoniae* also remodels its LPS lipid A domain to counteract CAMPs and polymyxins (Llobet *et al.*^2011^, ^2015^; Kidd *et al.*^2017^; Mills *et al.*^2017^). *Klebsiella pneumoniae* lipid A can be decorated with palmitate, 4-amino-4-deoxy-L-arabinose, phosphoethanolamine and 2-hydroxymyristate (Llobet *et al.*^2011^, ^2015^; Kidd *et al.*^2017^). These decorations provide resistance to CAMPs, and *K. pneumoniae* mutants lacking these lipid A decorations are attenuated for virulence in the mouse pneumonia model (Llobet *et al.*^2011^, ^2015^; Kidd *et al.*^2017^). There are reports showing that the lipid A acylation also mediates resistance to CAMPs (Clements *et al.*^2007^; Mills *et al.*^2017^). However, this role could be indirect since mutants deficient in the late acyltransferases *lpxM* and *lpxL* display changes in the CPS levels and the 2-hydroxylation of the lipid A, respectively (Clements *et al.*^2007^; Mills *et al.*^2017^).

The lipid A of *K. pneumoniae* shows a remarkable plasticity. Just a brief incubation with CAMPs upregulates the expression of the loci required to modify the lipid A with a concomitant increase in the lipid A species containing such modifications (Llobet *et al.*^2011^). The regulatory network controlling these transcriptional changes is complex and involves, at least, the PhoPQ, PmrAB and the Rcs systems (Llobet *et al.*^2011^). Of note, and in contrast to *Salmonella*, PhoPQ and PmrAB control independently the regulatory architecture governing these CAMPs-induced changes (Llobet *et al.*^2011^). Intriguingly, OmpA is also part of the regulatory network controlling systems required to ameliorate CAMPs bactericidal action (Llobet *et al.*^2009^). Whether there is any connection between the OmpA and the PhoPQ and PmrAB-regulated networks is currently unknown.

### Counteracting immune cells

Although it is well appreciated that the airway epithelium plays a central role orchestrating pulmonary inflammatory and immune responses against infections (Whitsett and Alenghat 2015), few studies detail the interaction of *Klebsiella* with these cells. Initial research argued that *Klebsiella* entry into epithelial cells may protect the pathogen from the actions of antibiotics and the immune system (Sahly *et al.*^2000^). These studies clearly demonstrated the role of the CPS limiting the attachment and internalisation to epithelial cells. However, subsequent studies have shown that epithelial cells engulfment of *Klebsiella* is most likely a host defence mechanism (Cortes *et al.*^2002^; Cano *et al.*^2009^). This interpretation is consistent with the observations that bacterial internalisation triggers an inflammatory response due to the activation of NF-κB signalling (Regueiro *et al.*^2006^), and that *K. pneumoniae* triggers a cytotoxic effect on epithelial cells (Cano *et al.*^2009^; Leone *et al.*^2016^). *Klebsiella*-induced cytotoxicity requires the presence of live bacteria and of CPS since it is observed with isolates expressing different amounts of CPS and/or different serotypes but not with non-capsulated bacteria (Cano *et al.*^2009^). *K. pneumoniae* infection also increases the levels of TLR4 and TLR2 in human airway epithelial cells which results in enhanced inflammatory response upon stimulation with TLR agonists (Regueiro *et al.*^2009^). TLR upregulation upon infection is dependent on the activation of NF-κB-governed signalling pathway by the CPS (Regueiro *et al.*^2009^). Evidence demonstrates that *Klebsiella* CPS is sensed by TLR4 (Regueiro *et al.*^2009^; Yang *et al.*^2011^; Frank *et al.*^2013^).

There is scarce data on the interaction between *Klebsiella* and neutrophils, although the recruitment of neutrophils to the lung is one of the hallmarks of *Klebsiella*-triggered pneumonia. *In vitro* experiments indicate that neutrophil-dependent clearance of *Klebsiella* occurs after phagocytosis. In turn, CPS abrogates killing by neutrophils due to its anti-phagocytic activity (Regueiro *et al.*^2006^; Pan *et al.*^2011^). Not surprisingly, antibodies against the CPS empower neutrophil-mediated killing (Diago-Navarro *et al.*^2018^), showing promise as new therapeutics to treat *Klebsiella* infections. Interestingly, *K. pneumoniae* does not induce NETosis (Branzk *et al.*^2014^), although anti-CPS antibodies enhance the release of neutrophil extracellular traps (NETs), which may contribute to extracellular killing of *Klebsiella* (Diago-Navarro *et al.*^2017^, ^2018^). NETs are large, extracellular, web-like structures composed of decondensed chromatin and neutrophil antimicrobial factors. NETs trap and kill a variety of microbes (Amulic *et al.*^2012^). NETs are released primarily via a cell death program that requires ROS, and the granule proteins myeloperoxidase and neutrophil elastase (Amulic *et al.*^2012^). Recently, it has been shown that *K. pneumoniae* interferes with clearance of neutrophils by efferocytosis (Jondle *et al.*^2018^). Efferocytosis is a regulated process which facilitates the elimination of apoptotic neutrophils by phagocytic cells, mainly macrophages, which prevents heightened inflammation triggered by dead cells (Ariel and Serhan 2012; Poon *et al.*^2014^; Angsana *et al.*^2016^). Mechanistically, *Klebsiella* infection of neutrophils results in a drastic decrease in the exposure of phosphatidylserine, which is recognised as ‘eat-me’ signal by macrophages to initiate the engulfment of neutrophils (Jondle *et al.*^2018^). The *Klebsiella* factor(s) responsible for this phenotype is currently unknown. Interestingly, evidence suggests that *Klebsiella* not only limits the efferocytosis of neutrophils, but it may also programme their cell death towards necroptosis (Jondle *et al.*^2018^). In contrast to apoptosis, necroptosis is a cell death triggering inflammation (Pasparakis and Vandenabeele 2015). Therefore, *Klebsiella-*triggered necroptosis may also contribute to the exuberant inflammation observed in this infection. It is then tempting to speculate that inhibition of necroptosis may improve *Klebsiella*-induced immunopathology. Preliminary observations indicate that this might be the case (Jondle *et al.*^2018^).

The specific role of dendritic cells in the clearance of *K. pneumoniae* has been poorly characterised, although there are data indicating that *Klebsiella* may activate different subsets of dendritic cells (Hackstein *et al.*^2013^). An outer membrane fraction of *K. pneumoniae*, containing CPS, LPS and porins, induces dendritic cell maturation (Van Elssen *et al.*^2010^). However, *K. pneumoniae* CPS and LPS attenuate dendritic maturation by hampering bacterial binding and internalisation (Evrard *et al.*^2010^). It should be mentioned that the latter results were obtained testing UV-killed bacteria. This technical approach is useful to assess the impact of an intact bacterial surface on cell activation/maturation but hampers any investigation on anti-immune systems deployed by live bacteria such as the injection of effector proteins by secretion systems. Nonetheless, future investigations are warranted to deconstruct the interaction between dendritic cells and *K. pneumoniae* to address whether the pathogen is able to survive intracellularly, the impact of *Klebsiella* infection on signalling governing cell maturation and whether *Klebsiella* may interfere with the processing of antigens and presentation of antigen-derived peptides to T cells, among other questions.

Historically, *K. pneumoniae* is considered an extracellular pathogen, yet there are reports suggesting that *K. pneumoniae* can survive in macrophages (Willingham *et al.*^2009^; Greco *et al.*^2012^; Fevre *et al.*^2013^; Fodah *et al.*^2014^). Providing compelling evidence for this hypothesis, Cano and co-workers (Cano *et al.*^2015^) have demonstrated that *K. pneumoniae* survives within human and mouse macrophages in a unique vacuolar intracellular compartment which deviates from the canonical endocytic pathway and it does not fuse with lysosomes. Furthermore, data suggest that *K. pneumoniae* triggers a programmed cell death in macrophages displaying features of apoptosis 10 h post infection (Cano *et al.*^2015^). One of the hallmarks of the *Klebsiella* containing vacuole (KCV) is its acidic pH, and, likewise for other pathogens (Yu *et al.*^2010^; Martinez, Siadous and Bonazzi 2018), phagosome acidification is essential for the intracellular survival of *K. pneumoniae* (Cano *et al.*^2015^). *Klebsiella pneumoniae* targets the PI3K–AKT–Rab14 axis to control phagosome maturation to survive inside macrophages (Cano *et al.*^2015^), strategy shared with *S. typhimurium* and *Mycobacterium tuberculosis* (Kuijl *et al.*^2007^), two classical intracellular pathogens. Interestingly, this axis is suitable for therapeutic manipulation to develop new anti-infective drugs. AKT inhibitors have already proven useful to eliminate intracellular *Salmonella* and *M. tuberculosis* (Kuijl *et al.*^2007^; Lo *et al.*^2014^), suggesting these inhibitors might provide selective alternatives to manage *K. pneumoniae* infections. Providing initial support to this hypothesis, *in vitro* experiments showed that AKT inhibition abrogates *Klebsiella* intracellular survival (Cano *et al.*^2015^). At present, the *Klebsiella* factors interfering with the phagosomal maturation pathway are unknown. Unexpectedly, the CPS does not play a large role, if any, in intracellular survival of *Klebsiella* as a *cps* mutant does not display any loss of viability upon phagocytosis (Cano *et al.*^2015^). In fact, *Klebsiella* downregulates the expression of the *cps* once inside the KCV (Cano *et al.*^2015^). It is tempting to speculate that *Klebsiella* may downregulate capsule expression to better survive in the intracellular environment which is poor in nutrients or because CPS may interfere with other factors required to manipulate phagosome maturation, among other possibilities.

Despite the crucial role of macrophages to clear *Klebsiella* infections *in vivo*, the macrophage responses following *Klebsiella* infection are poorly characterised. This is particularly relevant considering the plasticity of these cells, which can adjust their phenotype and physiology in response to environmental cues (Lavin *et al.*^2014^; Davies and Taylor 2015; Liddiard and Taylor 2015). These functional phenotypes led to classify macrophages as either classically activated M1 macrophages or alternatively activated M2 macrophages (Mills *et al.*^2000^; Murray *et al.*^2014^). M1 phenotype is characterised by the expression of high levels of pro-inflammatory cytokines, high production of reactive oxygen intermediates and iNOS-dependent reactive nitrogen intermediates, and promotion of Th1 response by IL12 production (Mills *et al.*^2000^; Murray *et al.*^2014^). The released cytokines and chemokines play a crucial role dictating cell-to-cell communications, hence regulating global tissue responses to infection. M2 macrophages are characterised by little to no secretion of pro-inflammatory cytokines, increased secretion of anti-inflammatory cytokines, enhanced scavenging of cellular debris, and promotion of tissue remodelling and repair (Mills *et al.*^2000^; Murray *et al.*^2014^). M1 macrophages are generally considered responsible for resistance against intracellular pathogens. However, uncontrolled M1 responses associated with acute infections may lead to immunopathology (Benoit, Desnues and Mege 2008). The M1-M2 transition may provide protection against overwhelming inflammation; however, a phenotypic switch may also favour pathogen survival. Indeed, a growing number of studies show that some pathogens have evolved different strategies to interfere with M1 polarisation, whereas chronic evolution of infectious diseases is thought to be associated with macrophage reprogramming toward a M2 profile (Benoit, Desnues and Mege 2008). Of note, reports exist showing the presence of M2 macrophages in *Klebsiella*-infected mouse models recapitulating human diseases, and the improvement in bacterial clearance when this macrophage population is eliminated *in vivo* (Dolgachev *et al.*^2014^; Ohama *et al.*^2015^; Tsuchimoto *et al.*^2015^). These observations suggest that *K. pneumoniae* might induce the polarisation of macrophages towards an M2-like phenotype. This hypothesis is initially supported by the fact that macrophages expressing high levels of IL10, a classical marker of M2 macrophages, are required to establish a macrophage/monocyte polarising tissue microenvironment (Fevre *et al.*^2013^). Conversely, treating mice with GM-CSF to stimulate M1 polarisation enhances *K. pneumoniae* clearance *in vivo* (Standiford *et al.*^2012^).

### Ablating host defence signalling

Early studies showed that *Klebsiella*-triggered pneumonia is characterised by high levels of the anti-inflammatory cytokine IL10 (Yoshida *et al.*^2000^, 2001). This cytokine is expressed by many cells of the immune system, and impacts on many cell types controlling the activation of innate immune responses (Gabrysova *et al.*^2014^). The induction of the anti-inflammatory response is mediated through the IL-10 receptor (IL-10R) and activation of signal transducer and activator of transcription 3 (STAT3) (Gabrysova *et al.*^2014^). The facts that neutralisation of IL10 *in vivo* results in prolonged survival of *Klebsiella*-infected mice with an enhancement of bacterial clearance in the lungs and blood, and an upregulation of inflammation (Greenberger *et al.*^1995^) suggests that *Klebsiella* exploits IL10 to attenuate immune responses. In agreement with this idea, IL10 overexpression causes a more pronounced bacteraemia and accelerated mortality in intratracheally infected mice (Dolgachev *et al.*^2014^). In turn, IFNγ plays an important role counteracting *Klebsiella*-induced IL10-dependent immune evasion because production of IL10 is significantly upregulated in infected *IFNγ^−^^/^^−^* with a concomitant increase in bacterial burden and decreased in inflammatory mediators (Moore *et al.*^2002^). Collectively, this evidence strongly supports the notion that induction of IL10 is part of the arsenal of *K. pneumoniae* immune evasion strategies. Adding additional weight to this notion, IL10 production is associated with *K. pneumoniae* pathogenicity because high levels of IL10 are only detected in mice infected with the wild-type strain but not in those mice infected with a *cps* mutant (Yoshida *et al*. 2001; Lawlor, Handley and Miller 2006). These results may suggest that the CPS is necessary for induction of IL10, although this has not been rigorously addressed yet. The identification of the cellular source of IL10 *in vivo*, the signalling pathway controlling *Klebsiella* induction of IL10 and the bacterial factor(s) needed for induction of IL10 are questions warranting future investigations.

*In vitro* and *in vivo* evidence demonstrates that a significant number of anti-*Klebsiella* responses are controlled by the transcriptional factor NF-κB upon activation of a TLR4/2-MyD88 signalling pathway (Regueiro *et al.*^2006^; Moranta *et al.*^2010^; Wieland *et al.*^2011^; Frank *et al.*^2013^; Tomas *et al.*^2015^). By limiting *Klebsiella* internalisation by epithelial cells, the CPS limits the activation of NF-κB and, hence, the production of IL8, ICAM1 and human defensins (Regueiro *et al.*^2006^; Moranta *et al.*^2010^). The reduced production of defensins by epithelial cells following *Klebsiella* infection can be considered another mechanism of resistance against these antimicrobial agents. However, *Klebsiella* also actively supresses NF-κB signalling by hijacking the host deubiquitinase cylindromatosis (CYLD) (Regueiro *et al.*^2011^; Frank *et al.*^2013^). CYLD deconjugates K63-linked ubiquitin chains to factors of the NF-κB signalling pathway, thereby abrogating the activation of the NF-κB signalling pathway (Sun 2008). In *cyld* knock-down cells, *Klebsiella* is no longer able to blunt the activation of TLR4/2-MyD88, leading to the production of IL8 following *Klebsiella* infection (Frank *et al.*^2013^). To upregulate the levels of CYLD, *K. pneumoniae* activates a NOD1 and an EGF receptor (EGFR)-phosphatidylinositol 3-OH kinase (PI3K)-AKT-PAK4-ERK-GSK3β signalling pathways (Regueiro *et al.*^2011^; Frank *et al.*^2013^). To the best of our knowledge, *K. pneumoniae* is the first pathogen to date activating a NLR receptor to blunt inflammation. The activation of NOD1 is mediated by the inhibition of the Rho GTPase Rac1, although the *Klebsiella* factor(s) responsible is unknown (Regueiro *et al.*^2011^). Whilst other bacterial pathogens are known to activate EGFR (Zhang *et al.*^2004^; Keates *et al.*^2007^; Choi *et al.*^2011^; Xu *et al.*^2011^), only *Klebsiella* manipulates this receptor to ablate the production of inflammatory cytokines. *Klebsiella*-dependent activation of the EGFR pathway is mediated by the CPS and, therefore, a *cps* mutant does not activate EGFR (Frank *et al.*^2013^). Interestingly, CPS-mediated EGFR activation is indirect and requires the cSRC kinase, which is activated upon recognition of the CPS by TLR4 (Frank *et al.*^2013^). NOD1 and EGFR do not play redundant roles in *Klebsiella*-triggered block of NF-κB activation because in cells in which EGFR and NOD1 expressions are downregulated by siRNA the anti-inflammatory effect is completely abolished in contrast to what happens in the single knockdown cells (Regueiro *et al.*^2011^; Frank *et al.*^2013^).

The production of inflammatory mediators and defensins by epithelial cells following *Klebsiella* infection is also governed by the MAPKs p38, ERK and JNK (Wu *et al.*^2006^; Moranta *et al.*^2010^; Regueiro *et al.*^2011^). *Klebsiella* inhibits MAPKs activation via the MAPK phosphatase-1 (MKP-1) (Regueiro *et al.*^2011^). *Klebsiella* upregulates the levels of MKP-1 by activating NOD1, and both MKP-1 and CYLD play synergistic roles to abrogate the production of IL8 by infected epithelial cells (Regueiro *et al.*^2011^). It is remarkable that *Klebsiella* hijacks two host proteins, CYLD and MKP-1, involved in immune homeostasis after inflammation to protect the host from an overwhelming inflammatory response (Liu, Shepherd and Nelin 2007; Sun 2008). This immune evasion strategy is radically different to that deployed by other pathogens based on exploiting bacterial effectors to ablate NF-κB and MAPKs activation.

As a result of a high-throughput screen interrogating a library of 5320 *K. pneumoniae* transposon mutants using as a readout a gain of NF-κB activation (Tomas *et al.*^2015^), 114 mutants no longer blunting NF-κB signalling were identified. Metabolism and envelope-related genes are the gene ontology categories accounting for half of the loci identified in the screening (Tomas *et al.*^2015^). Follow-up characterisation conclusively established that the LPS O-polysaccharide and the pullulanase (PulA) type 2 secretion system (T2SS) are required for full effectiveness of the immune evasion (Tomas *et al.*^2015^). Importantly, the CPS, the LPS O-polysaccharide and the PulA T2SS do not play a redundant role attenuating inflammation (Tomas *et al.*^2015^). In good agreement with the *in vitro* results, the LPS O-polysaccharide and *pulA* mutants induce higher inflammation *in vivo* than the wild-type strain (Tomas *et al.*^2015^). Furthermore, they are attenuated in the pneumonia mouse model (Tomas *et al.*^2015^). The fact that LPS O-polysaccharide and T2SS mutant-induced responses are dependent on TLR2-TLR4-MyD88 activation suggests that LPS O-polysaccharide and PulA perturb TLR-dependent recognition of *K. pneumoniae*.

OmpA has also been shown to be implicated in preventing TLR activation to limit inflammatory responses *in vitro* and *in vivo* (March *et al.*^2011^). The contribution of OmpA to *Klebsiella* immune evasion is independent of CPS because a double mutant lacking *cps* and *ompA* induces higher inflammatory response than any of the single mutants (March *et al.*^2011^). An *ompA* mutant is also attenuated in the pneumonia mouse model (March *et al.*^2011^), further highlighting the importance of immune evasion for *Klebsiella* virulence. However, studies testing OmpA purified from *K. pneumoniae* have yielded opposite results. Purified OmpA activates TLR2 signalling leading to enhanced cytokine production (Jeannin *et al.*^2000^; Soulas *et al.*^2000^; Pichavant *et al.*^2003^), and instillation of the purified protein *in vivo* also results in upregulation of inflammation (Jeannin *et al.*^2000^; Soulas *et al.*^2000^; Pichavant *et al.*^2003^). Of note, an *E. coli ompA* mutant strain induces higher expression of pro-inflammatory mediators (Selvaraj and Prasadarao 2005), strongly suggesting that indeed OmpA plays a role in immune evasion. The contradiction between the results observed assessing the role of OmpA in the biological context of the bacterial membrane and those testing the protein as ligand reflects the importance of investigating the interplay between pathogens and the immune system interrogating whole live bacteria instead of purified ligands.

### Subversion of nutritional immunity

To limit infections, humans and other mammals restrict access to essential metals in a process termed ‘nutritional immunity’. Originally referring to restriction of iron availability, the term now also applies to mechanisms for withholding other metals in addition to Fe such as Zn, Mn and Cu, or directing the toxicity of these metals against pathogens (Palmer and Skaar 2016). In the case of *Klebsiella* infections, most of the research has focused on understanding how *Klebsiella* subverts iron nutritional immunity. Like many other *Enterobacteriaceae, Klebsiella* secretes the siderophore enterobactin to compete iron off of iron-loaded host proteins (Wooldridge and Williams 1993). Notably, and as a perfect example of a host–pathogen arms race, the host employs lipocalin 2 to compete out bacteria from binding the siderophore (Bachman, Miller and Weiser 2009; Bachman *et al.*^2011^, ^2012^). It is then not surprising that *Klebsiella* strains secrete additional siderophores, namely aerobactin, salmochelin and yersiniabactin (Bachman *et al.*^2011^; Russo *et al.*^2011^, ^2014^). Yersiniabactin promotes *Klebsiella* pneumonia by evading lipocalin 2 (Lawlor, O’connor and Miller 2007; Bachman *et al.*^2011^). Furthermore, epidemiological studies demonstrate that the acquisition of yersiniabactin is one of the traits of *Klebsiella* strains causing invasive infections (Bachman *et al.*^2011^; Holt *et al.*^2015^), highlighting the importance of overcoming nutritional immunity in *Klebsiella* infection biology. It is interesting to consider the regulation of the expression of these siderophores during infection but also in the context of survival in the environment. Initial findings suggest that enterobactin plays a major role under more iron-restricted conditions than any of the other siderophores (Lawlor, O’connor and Miller 2007).

Intriguingly, recent evidence argues that in *Klebsiella*-triggered pneumonia siderophores contribute to bacteria dissemination by stabilising the transcriptional factor HIF-1α (Holden *et al.*^2016^). This transcriptional factor governs mucosal immunity and cellular intrinsic immunity (Palazon *et al.*^2014^). However, in the case of *Klebsiella* infections the data are consistent with the hypothesis that the pathogen exploits HIF-1α to promote infection (Holden *et al.*^2016^). Further studies are warranted to validate this hypothesis, and to identify the HIF-1α-governed responses facilitating *Klebsiella* dissemination.

## ANTIBIOTIC RESISTANCE AND *KLEBSIELLA* IMMUNE EVASION

Whilst the impact of antibiotic resistance in bacterial physiology is well appreciated, it is still poorly understood the relationship between virulence and antibiotic resistance in the absence of antibiotic challenge, and whether antibiotic resistance mechanisms affect the interaction of pathogens with innate host defence mechanisms. In the case of *Klebsiella* infections, recent studies have investigated these questions by comparing infection outcomes between strains with different patterns of antibiotic resistance. For example, the inflammatory responses induced by one strain of each of the two clades of the globally disseminated *K. pneumoniae* ST258 clonal group have been compared. Preliminary results might support that one of the clades triggers more inflammation than the other (Castronovo *et al.*^2017^; Clemente *et al.*^2017^). The ability of few clinical isolates of the same clonal group to avoid phagocytosis by neutrophils and to survive in serum has been also investigated (Kobayashi *et al.*^2016^; DeLeo *et al.*^2017^). However, the number of strains tested is small and it is not evident how representative these strains are. Furthermore, there is limited information on the *Klebsiella* factor(s) responsible for the phenotypes described. One possible explanation is the challenge to construct mutants in multidrug-resistant clinical isolates. This is exemplified in the recent work from the Prince laboratory in which authors investigated the evolution of a local outbreak of a clone of the ST258 group (Ahn *et al.*^2016^). The locally predominant clone, represented by strain KP35, outcompeted related ST258 strains by becoming more competent supressing inflammatory responses (Ahn *et al.*^2016^). Mechanistically, KP35 triggered the recruitment to the lung of Ly6C^hi^ monocytic myeloid-derived suppressor cells that lacked phagocytic capabilities and contributed to create an anti-inflammatory microenvironment (Ahn *et al.*^2016^). Authors identified the acquisition of four new orthologues by KP35 in comparison to other ST258 strains (Ahn *et al.*^2016^), suggesting that the acquisition of these novel genes contributed to its fitness and persistence. Unfortunately, the lack of genetic tools to manipulate KP35 made impossible to pinpoint any of the genetic changes as responsible for the enhanced fitness *in vivo*. Nonetheless, this study demonstrates how the challenge imposed by the immune system drives the adaptation of strains to the host microenvironment enhancing fitness independently of the antimicrobial selective pressure.

Few studies have addressed the contribution of antibiotic resistance mechanisms to virulence in the absence of antibiotic pressure. However, the emerging scenario supports a strong relationship between immune evasion, virulence and antibiotic resistance mechanisms in *K. pneumoniae*. OmpK35 and OmpK36 are two porins whose expression is downregulated in many clinical isolates including ESBL-producing and carbapenem-resistant strains (Ardanuy *et al.*^1998^; Hernandez-Alles *et al.*^1999^, ^2000^; Mena *et al.*^2006^; Shin *et al.*^2012^). Downregulation of OmpK35 and OmpK36 limits the influx of antibiotics to the bacterium (Ardanuy *et al.*^1998^; Hernandez-Alles *et al.*^1999^, ^2000^; Mena *et al.*^2006^; Shin *et al.*^2012^), whereas restoration of expression decreases antibiotic resistance (Martinez-Martinez *et al.*^1999^; Doumith *et al.*^2009^; Tsai *et al.*^2011^). OmpK36 contributes to *Klebsiella* virulence in the peritonitis and pneumonia mouse model (Chen *et al.*^2010^; Tsai *et al.*^2011^; March *et al.*^2013^). Mechanistically, OmpK36 is not required for serum resistance (Tsai *et al.*^2011^), and does not play any role counteracting the bactericidal action of CAMPs (Llobet *et al.*^2009^). However, OmpK36 prevents phagocytosis by neutrophils and alveolar macrophages (Tsai *et al.*^2011^; March *et al.*^2013^) which may explain the attenuation of the *ompK36* mutant. Interestingly, the antiphagocytic effect of OmpK36 is also observed in the absence of CPS because a double mutant lacking *cps* and *ompK36* is phagocytosed in higher numbers than the single mutants (March *et al.*^2013^). Whether OmpK36 or any other porin shapes the inflammatory response following *Klebsiella* infection warrants investigation.

AcrAB is an efflux pump implicated in antibiotic resistance in *K. pneumoniae* and other *Enterobacteriaceae* (Li, Plesiat and Nikaido 2015). Increased expression of AcrAB contributes to resistance against several antibiotics in clinical isolates (Bialek-Davenet *et al.*^2015^; Wang *et al.*^2015^) which is consistent with the wide range of substrates that this pump transports (Li, Plesiat and Nikaido 2015). However, AcrAB is also necessary for *Klebsiella* virulence because an *acrAB* mutant is attenuated in the pneumonia mouse model (Padilla *et al.*^2010^). Moreover, the *acrAB* mutant is more susceptible to CAMPs present in human bronchoalveolar lavage fluid and to human antimicrobial peptides than the wild-type strain (Padilla *et al.*^2010^), demonstrating a role for this efflux pump counteracting innate defences in addition to contributing to a multidrug resistance phenotype.

Several regulators have been demonstrated to influence *acrAB* expression in *Klebsiella* and other *Enterobacteriaceae* (Weston *et al.*^2017^). For example, the AraC family transcriptional regulator RamA is involved in tigecycline resistance via upregulation of AcrAB (Rosenblum *et al.*^2011^). This antibiotic has been introduced into clinical practice for the treatment of community-acquired Gram-negative infections caused by extended-spectrum β-lactamase-producing *Enterobacteriaceae*. Interestingly, RamA overexpression, a feature observed in clinical isolates even before the introduction of tigecycline (Rosenblum *et al.*^2011^), results in a multidrug-resistant strain with enhanced virulence (De Majumdar *et al.*^2015^). Increased levels of RamA decrease susceptibility of *Klebsiella* to polymyxins and the human CAMP LL-37, and reduce bacterial adhesion and uptake into macrophages (De Majumdar *et al.*^2015^). Infection with a strain overexpressing RamA results in higher bacterial burden in the lungs and increase systemic dissemination (De Majumdar *et al.*^2015^), demonstrating the relevance of these phenotypes to heightened fitness *in vivo*. RamA overexpression triggers significant changes in *Klebsiella* transcriptome making then difficult the identification of the RamA-controlled loci responsible for the increase virulence (De Majumdar *et al.*^2015^). Initial experimental evidence demonstrates that RamA directly activates *acrAB* and loci implicated in lipid A remodelling (De Majumdar *et al.*^2015^). Based on the discussed evidence, it is tempting to postulate that these loci may underline RamA-linked phenotypes.

*lpxO* is one of the lipid A loci regulated by RamA (De Majumdar *et al.*^2015^). LpxO mediates the 2-hydroxylation of the lipid A in *Klebsiella* and other Gram-negative pathogens including *Salmonella* and *Pseudomonas* (Gibbons *et al.*^2000^, ^2008^; Moskowitz and Ernst 2010). Remarkably, *K. pneumoniae* switches on this lipid A modification in the lungs of infected mice in a PhoPQ-dependent manner (Llobet *et al.*^2015^). This modified lipid A does not activate inflammatory responses *in vivo* and *in vitro* to the same extent as the lipid A produced by *Klebsiella* in normal laboratory medium (Llobet *et al.*^2015^), demonstrating that the 2-hydroxylation of the lipid A contributes to immune evasion. Not surprisingly, an *lpxO* mutant is attenuated in the pneumonia mouse model (Insua *et al.*^2013^; Llobet *et al.*^2015^). Furthermore, this lipid A modification also mediates resistance to human antimicrobial peptides and colistin (Llobet *et al.*^2015^; Mills *et al.*^2017^), one of the last options to treat multidrug-resistant *Klebsiella* (Li *et al.*^2006^). Changes in the lipid A acylation pattern have also been linked to resistance to colistin and virulence independently of the lipid A decorations (Clements *et al.*^2007^; Halaby *et al.*^2016^; Mills *et al.*^2017^).

Resistance to colistin is of significant concern in those settings with high number of ESBL and carbapenem-producing *Klebsiella* strains. Unfortunately, colistin resistance arises frequently upon treatment and even following decontamination protocols to limit infections in the healthcare setting. Several recent studies highlight the emergence of colistin resistance in multidrug-resistant *K. pneumoniae* arising from loss of function mutations of the *mgrB* gene, a negative regulator of the PhoPQ signalling system (Lippa and Goulian 2009; Cannatelli *et al.*^2013^, ^2014^). Alarmingly, *mgrB*-dependent colistin resistance is not associated with any fitness cost *in vitro* and *in vivo* and it is stably maintained in the absence of selective pressure (Cannatelli *et al.*^2015^; Kidd *et al.*^2017^). This may explain the rapid dissemination of strains carrying this resistance mechanism in the clinical setting. Further complicating the health problem posed by these infections, inactivation of *mgrB* enhances *K. pneumoniae* virulence (Kidd *et al.*^2017^). In fact, the heightened virulence of these strains might be one of the explanations underlying the increased mortality associated with these infections (Capone *et al.*^2013^; Falcone *et al.*^2016^). Mechanistically, *mgrB* mutation induces PhoPQ-governed lipid A remodelling which confers not only resistance to polymyxins, but also enhances *K. pneumoniae* virulence by decreasing CAMPs susceptibility and attenuating the activation of early host defence responses in macrophages (Kidd *et al.*^2017^). These findings also illustrate that the development of antibiotic resistance is not inexorably linked with subdued bacterial fitness and virulence. Overall, this research stresses the importance of considering antimicrobial resistance and virulence together, and the urgent need to include the identification of virulent clones in clinical microbiology laboratories.

## CONCLUSIONS AND PERSPECTIVES

The World Health Organization has recently included *Klebsiella* in the critical list of microorganisms for which new therapeutics are urgently needed. The increasing isolation of strains resistant to ‘last resort’ antimicrobials has significantly narrowed, or in some settings completely removed, the therapeutic options for the treatment of *Klebsiella* infections. Not surprisingly, several international organisations including the United Nations have regarded multidrug-resistant *Klebsiella* as an ‘urgent threat to human health’. Whilst there are several new therapeutic approaches under investigation including the use of bacteriophages, enzybiotics (phage-derived lytic enzymes) and antibodies against *Klebsiella* surface molecules; unfortunately, at present, we cannot identify candidate compounds in late-stage development for treatment of multidrug *Klebsiella* infections. This pathogen is exemplary of the mismatch between unmet medical needs and the current antimicrobial research and development pipeline. Furthermore, our understanding of *Klebsiella* pathogenesis still contains considerable gaps. Therefore, understanding the various Achilles heels of host defence against *Klebsiella* is a high priority and timely for the development of preventative and novel therapeutic measures to combat these infections.

*K. pneumoniae* has been traditionally considered a formidable pathogen evading defence mechanisms. The roles of CPS limiting the activation of inflammatory responses, preventing the bactericidal action of complement and CAMPs, and abrogating phagocytosis by neutrophils and macrophages are perfect examples of *Klebsiella* stealth strategies. The lack of expression of porins to avoid complement activation, and the role of the LPS O-polysaccharide to limit complement deposition on the bacterial surface are other examples of this *Klebsiella* stealth behaviour.

However, a body of evidence mostly recently obtained strongly suggests that *Klebsiella* has also evolved mechanisms to actively supress innate immune responses, illustrating the diversity and sophistication of *Klebsiella* immune evasion strategies. The manipulation of phagosome maturation to establish the KCV, the upregulation of lipid A decorations upon sensing the host microenvironment to counteract the action of CAMPs and to avoid the activation of NF-κB-controlled inflammation are examples of these *Klebsiella* subversion strategies. At the cellular level, the evidence clearly demonstrates that an essential aspect of *K. pneumoniae* infection biology is to thwart the TLR-dependent activation of host defence responses controlled by NF-κB and MAPKs. To do so, *Klebsiella* hijacks the deubiquitinase CYLD and the MAPKs phosphatase MKP-1. Both proteins play a pivotal role in host homeostasis by limiting overwhelming inflammatory responses to avoid immunopathology. This is an exquisite example of how a bacterial pathogen exploits the host machinery to avoid immune activation. This immune evasion strategy is different to those deployed by other bacterial pathogens such as *Listeria, Legionella, E. coli, Salmonella* or *M. tuberculosis* based on deploying bacterial proteins and effectors to blunt host defence signalling pathways. Considering the increasing number of host proteins contributing to balance host responses, it is tempting to speculate that *Klebsiella* may hijack other proteins as well. Interestingly, *Klebsiella* activates TLR4 and NOD1 signalling to increase the levels of CYLD and MKP-1. Thus, *Klebsiella* infection biology is largely based on the balance between avoiding PRR detection/activating PRR for immune evasion, hence being a true pathogen-host arms race. At the tissue level, *Klebsiella* exploits the anti-inflammatory properties of IL10 to shape the local microenvironment. Collectively, the data strongly suggest that *Klebsiella* hijacks host effectors devoted to prevent overwhelming inflammatory responses as virulence strategy.

The CPS is necessary but not sufficient for *Klebsiella* immune evasion, and *Klebsiella* exploits few other factors acting synergistically to abrogate immune responses. The importance of avoiding immune responses in *Klebsiella* pathogenesis is marked by the fact that mutants lacking these factors are attenuated *in vivo*. However, we believe there is much left to be uncovered about which *K. pneumoniae* factors are required during infection. In this regard, recent epidemiological studies have demonstrated the wide spectrum of genetic diversity within the genus (Holt *et al.*^2015^; MC Lam *et al.*^2018^). It is important to note that the epidemiological studies are biased towards strains of clinical origin. There is limited information on the attributes of strains isolated from the environment, and the potential transmission route between the environment, and the healthcare setting. The immune evasion strategies described so far rely on factors belonging to the *Klebsiella* core genome, indicating that *Klebsiella* anti-immunology is at the fulcrum of the species biology. However, the *Klebsiella* accessory genome is close to 30 000 genes, more than the human genome (Holt *et al.*^2015^). Moreover, around 50% of the genes of an individual strain belong to the accessory genome (Holt *et al.*^2015^), illustrating the considerable genomic plasticity that is contained within this species. Notably, there is virtually no information on the contribution of the accessory genome to *Klebsiella* immune evasion, although recent findings suggest that the accessory genome may also facilitate the adaptation of *Klebsiella* to host microenvironments (Ahn *et al.*^2016^). Future studies should address the relative contribution of the core and accessory genome to *Klebsiella* infection biology, and uncover the regulatory networks coordinating the spatial-temporal expression of these factors. Additionally, there is a significant knowledge gap on the virulence of other *Klebsiella* species including *K. oxytoca* and *K. variicola*. To obtain comprehensive information, it will be necessary to assess infection phenotypes interrogating large collection of strains from different environments and genomic backgrounds to capture *Klebsiella* diversity. Ideally, these studies will yield a catalogue of virulence factors and host pathways subverted across different infections and common to many strains, leading to the definition of the *Klebsiella* signature of infection. This knowledge may help to better stratify patients and to tailor treatments not only based on the antibiotic resistance profile but also taking into consideration virulence features of the infecting strain. Nonetheless, it remains a challenge to develop efficient genetic tools to construct mutants and to complement multidrug-resistant strains to provide mechanistic information on the specific factors responsible for the investigated virulence phenotypes.

It is also important to understand how *Klebsiella* evades the immunological challenges it faces when colonising/infecting the GI tract and the oropharyngeal sites. This may require developing new infection models to follow bacterial colonisation for at least several days. It is exciting to consider that the immune evasion strategies of *Klebsiella* may differ in different mucosae. There is much to be learnt about how *Klebsiella* disseminates from the primary infection site, either the lung or the gut, to other sites. For example, a yet poorly investigated question is the interaction between *Klebsiella* and endothelial cells lining vascular and lymphatic vessels. This interaction is clinically relevant considering the number of sepsis cases associated with *Klebsiella* infections (Girometti *et al.*^2014^). We do foresee that the interplay between *Klebsiella*-endothelial cells could be another host–pathogen battleground playing an important role in *Klebsiella* infection biology.

As mentioned above, a crucial virulence strategy of *Klebsiella* is to subdue TLR-mediated inflammatory responses. Substantial data also confirm the relative importance of IL23/IL17 and IL12/IFNγ-activated signalling pathways to generate an effective innate immune response against *Klebsiella*. It can be then argued that *Klebsiella* lack of immunostimulatory activities may limit the activation of these signalling axes. However, and considering the sophisticated strategies deployed by the pathogen to perturb TLR recognition and activation, we postulate that *Klebsiella* has devised specific strategies to attenuate IL23/IL17 and IL12/IFNγ signalling. Among other possibilities, it is tempting to speculate that *Klebsiella* may target the cells responsible for the production of these cytokines, ablate the signalling pathways needed for the production of these cytokines, and even subvert the receptors and signalling pathways activated by these cytokines. Future studies combining *in vivo* and *in vitro* approaches are warranted to investigate these questions.

The concept of targeted specific antibiotic therapy for *Klebsiella* infections is plausible with the availability of real-time PCR-based methods for rapid detection. This will vastly reduce the time required to direct appropriate therapy and limit the use of empirical broad range treatments. However, the global emergence of multidrug-resistant *Klebsiella* strains significantly reduces the available options to treat these infections, making it imperative to consider other options. It is evident that therapeutic strategies to improve innate immune mechanisms may enhance clearance of *Klebsiella*. This could be achieved through pro-inflammatory agents or by boosting TLR-governed defences, or by abrogating *Klebsiella* immune evasion strategies. In this context, it would appear that targeting major *Klebsiella* immune evasins such as the CPS could be a successful avenue to counteract *Klebsiella* anti-immune strategies. However, given the genetic diversity of this pathogen we consider likely the selection of clones able to evade this therapy focused on a single or even a limited number of gene products. Instead, we favour the approach of targeting the host factors manipulated by *Klebsiella* to subvert host defence responses (Zumla *et al.*^2016^). This host-directed therapeutics approach is thought to apply less selective pressure for the development of resistance than traditional strategies, which are aimed at killing pathogens or preventing their growth. Interestingly, there might be drugs already available targeting the host factors hijacked by *Klebsiella*, which might be even in clinical use to treat other diseases. From the drug discovery point of view, this significantly circumvents the drug development process, hence allowing a fast-track transition from pre-clinical research to clinical development.

Despite our significant advances on our understanding of *Klebsiella* pathogenesis during the last years, we still have a fragmented picture of the interaction of *K. pneumoniae* with the immune system, and there is a significant knowledge gap on the repertoire of virulence factors enabling *Klebsiella* to overcome defences to multiply in the tissues. Further studies on the fascinating infection biology of *Klebsiella* will help to shore-up more precisely the vulnerable hot spots of our immune system while uncovering new means exploited by a human pathogen to counteract the challenge of an activated immune system.
